# The Interaction of Human Glutathione Transferase GSTA1-1 with Reactive Dyes

**DOI:** 10.3390/molecules26082399

**Published:** 2021-04-20

**Authors:** Mohammed Hamed Alqarni, Ahmed Ibrahim Foudah, Magdy Mohamed Muharram, Nikolaos E. Labrou

**Affiliations:** 1 Department of Pharmacognosy, College of Pharmacy, Prince Sattam Bin Abdulaziz University, Alkharj 11942, Saudi Arabia; a.foudah@psau.edu.sa; 2 Department of Pharmaceutics, College of Pharmacy, Prince Sattam Bin Abdulaziz University, Alkharj 11942, Saudi Arabia; m.moharm@psau.edu.sa; 3 Department of Microbiology, College of Science, Al-Azhar University, Nasr City, Cairo 11884, Egypt; 4 Laboratory of Enzyme Technology, Department of Biotechnology, School of Food, Biotechnology and Development, Agricultural University of Athens, 75 Iera Odos Street, GR-11855 Athens, Greece

**Keywords:** anthraquinone, chemotherapy, enzyme inhibitor, glutathione transferase

## Abstract

Human glutathione transferase A1-1 (hGSTA1-1) contributes to developing resistance to anticancer drugs and, therefore, is promising in terms of drug-design targets for coping with this phenomenon. In the present study, the interaction of anthraquinone and diazo dichlorotriazine dyes (DCTD) with hGSTA1-1 was investigated. The anthraquinone dye Procion blue MX-R (PBMX-R) appeared to interact with higher affinity and was selected for further study. The enzyme was specifically and irreversibly inactivated by PBMX-R, following a biphasic pseudo-first-order saturation kinetics, with approximately 1 mol of inhibitor per mol of the dimeric enzyme being incorporated. Molecular modeling and protein chemistry data suggested that the modified residue is the Cys112, which is located at the entrance of the solvent channel at the subunits interface. The results suggest that negative cooperativity exists upon PBMX-R binding, indicating a structural communication between the two subunits. Kinetic inhibition analysis showed that the dye is a competitive inhibitor towards glutathione (GSH) and mixed-type inhibitor towards 1-chloro-2,4-dinitrobenzene (CDNB). The present study results suggest that PBMX-R is a useful probe suitable for assessing by kinetic means the drugability of the enzyme in future drug-design efforts.

## 1. Introduction

Glutathione transferases (GSTs) are phase II detoxifying enzymes that catalyze various detoxification reactions [1,2,3,4,5]. GSTs exist and function as homo- or heterodimer proteins. Each subunit has an active site consisting of a polar GSH-binding site (G-site) and an adjacent hydrophobic binding site for electrophilic substrates (H-site) [2,4,5].

The five isoenzymes (GSTA1–A5) of the alpha class are encoded by a gene cluster that contains five genes localized on chromosome 6p12. Human tissues widely express GSTA1, A2, and A4 transcripts, whereas expression of GSTA3 is rare, and GSTA5 has yet to be detected in human tissues [3,6,7]. The isoenzyme hGSTA1-1 is primarily expressed in the human liver, representing 2% of the total cytoplasmic proteins [3]. It is also found in the kidney and various other human tissues [3]. It has been extensively studied since it catalyzes the detoxification of a wide range of electrophilic compounds, including carcinogenic metabolites, environmental pollutants, polycyclic aromatic hydrocarbons and diol-epoxides. Among the electrophilic compounds that are detoxified by hGSTA1-1 are several alkylating chemotherapeutics that are used in the treatment of cancer (e.g., busulfan, chlorambucil, etc.) [8,9,10,11,12,13,14]. This phenomenon, in combination with the overexpression of GSTs in cancer cells, is associated with developing resistance to many anticancer drugs [14,15]. In addition, GSTs have been shown to contribute to resistance to chemotherapeutic agents by regulating cell signaling pathways that control cell proliferation, apoptosis and redox homeostasis [7,16,17,18]. Furthermore, hGSTA1-1 suppresses JNK signaling activation by proinflammatory cytokine and oxidative stress, suggesting a potential protective role of hGSTA1-1 against JNK [19]. These actions of hGSTA1-1 are of great significance since they may compromise the efficacy and, therefore, the successful outcome of therapeutic approaches. To this end, one of the strategies being developed to address this problem relies on applying GST-targeted inhibitors. Numerous compounds with different degrees of efficacy and inhibition potency have already been studied over the last years [7,11,12,13,14,15,20,21,22,23,24]. GST inhibitors are classified according to their binding specificity [1,5]. Those that bind to the G-site or H-site and those that bind to the ligandin-binding site (L-site). The existence of a distinct L-site has been confirmed in several GST isoenzymes, including hGSTA1-1 [25,26,27,28,29]. The L-site role remains obscure, although it has been suggested that is involved in the storage and rapid transport of lipophilic molecules in the aqueous phase of the cell.

In the present work, we investigate the interaction of a range of dichlorotriazine dyes with hGSTA1-1, aiming at assessing their ability to function as irreversible probes/inhibitors towards the enzyme. The results of the present work provide new insights into the specificity of the ligandin-binding site. The study’s outcome can facilitate a more rational approach for developing effective GST-targeted chemosensitizers for reversing anticancer drug resistance.

## 2. Results and Discussion

### 2.1. Screening of the Inhibition Potency of DCTD

**Figure 1:**
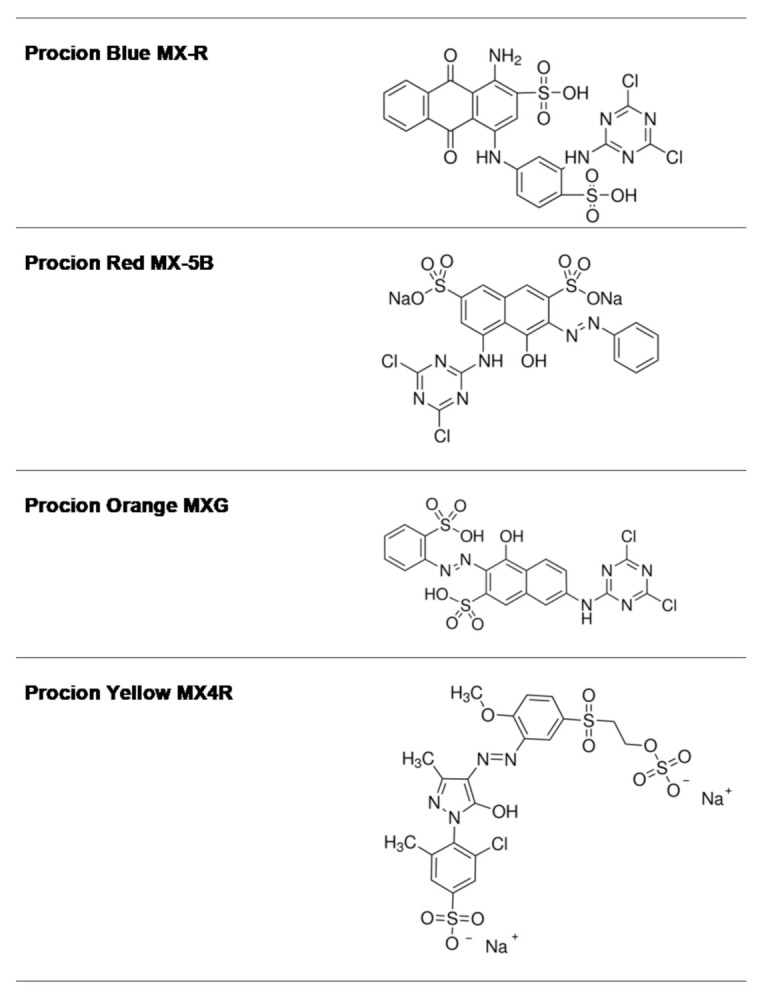
The structure of the anthraquinone and diazo dyes used in the present study.

**Figure 2:**
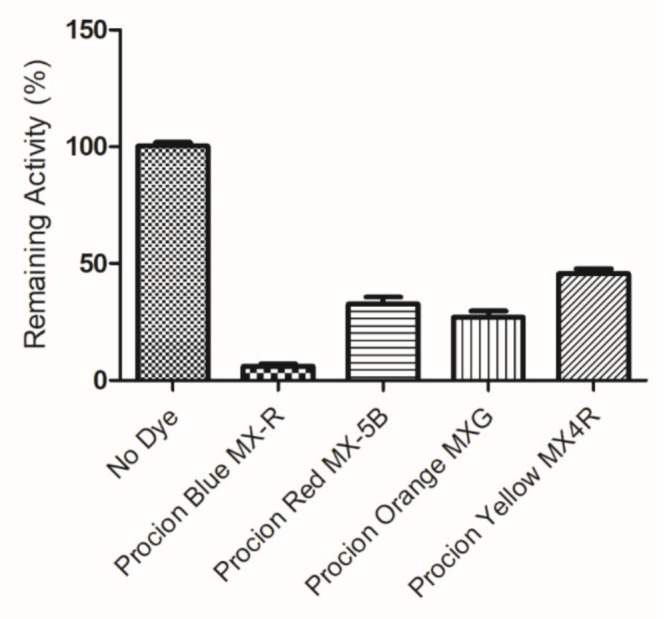
Screening of the inhibition potency of a range of selected diazo and anthraquinone dyes towards hGSTA1-1.

Reactive dyes are useful and versatile tools for probing structure–function relationships in various proteins, including GSTs [30,31,32,33]. Four widely used dyes that belong to the anthraquinone and diazo group (Figure 1) were screened for their ability to bind and inhibit the isoenzyme hGSTA1-1. The results showed (Figure 2) that the anthraquinone dye PBMX-R displayed a higher inhibition potency, and therefore, it was selected for further study. The ability of the anthraquinone dye Cibacron blue 3GA (CB3GA) to inhibit the activity of other GSTs has been previously investigated [31,34,35]. For example, CB3GA has been used as a diagnostic probe to classify the mouse GST isoenzymes [35]. In addition, the interaction of CB3GA with the isoenzyme hGSTP1-1, which is involved in developing resistance to several anticancer drugs, has been investigated by X-ray crystallography [34]. More recently, the interaction of CB3GA and its structural fragments with the isoenzyme *Sj*GST from *Schistosoma japonicum* was investigated [31].

The dye PBMX-R (Figure 1) possesses an electrophilic reactive dichlorotriazine moiety which is susceptible to chemical modification by several nucleophilic side chains in proteins. Therefore, it can react covalently with proteins and enzymes [30,31,32].

### 2.2. Inactivation of hGSTA1-1 by PBMX-R

**Figure 3:**
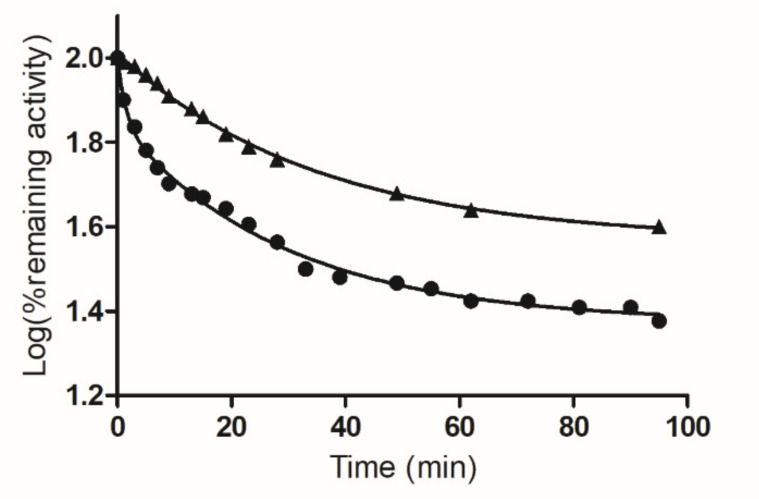
Time course of inactivation of hGSTA1-1 by PBMX-R. The effect of PBMX-R concentration (5.0 μM ●) on hGSTA1-1 activity. Time course of inactivation of hGSTA1-1 by 5.0 μM PBMX-R in the presence of S-pNb-GSH (1 mM ▲). At the times indicated, aliquots were withdrawn and assayed for enzymatic activity. Rate constants for the reaction exhibiting biphasic kinetics were calculated as described previously [8,31] using the computer program GraphPad Prism 5 software (San Diego, CA, USA). The data points show one representative experiment.

**Figure 4:**
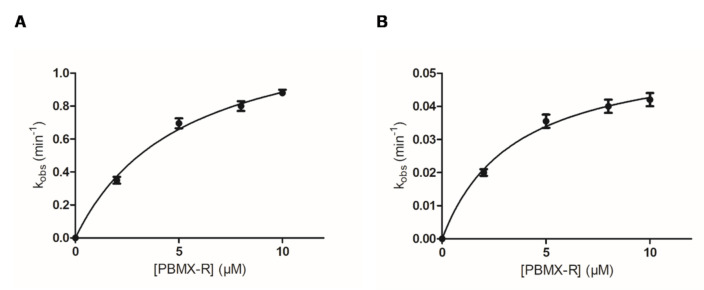
Dependence of the inactivation rate of hGSTA1-1 (*k_obs_*) on the PBMX-R concentration for the fast (**A**) and slow (**B**) phase of inactivation reaction. Kinetic parameters (*k_cat_* and *K_D_*) were calculated as described previously [8,31] using the computer program GraphPad Prism 5 software (San Diego, CA, USA). Data points represent mean values with error.

When hGSTA1-1 was incubated with PBMX-R, the enzyme activity was progressively diminished in a time (Figure 3) and concentration-dependent manner (Figure 4). Under identical conditions, the omission of PBMX-R from the incubation mixture results in loss of enzyme inactivation. Chromatography of the reaction mixture by gel filtration on Sephadex G-25 column did not recover the enzyme activity, indicating the formation of a covalent complex between hGSTA1-1/PBMX-R.

As shown in Figure 3, the inactivation reaction proceeds in two distinct phases: an initial fast phase of inactivation and a slow phase. Figure 4 shows the dependence of the inactivation rates for the fast and slow phases on the concentration of PBMX-R. Both phases display the characteristics of a chemical reaction since their progression depends on time, and for a certain time, it depends on the concentration of PBMX-R. The observed hyperbolic pattern (Figure 4) consisted of forming a Michaelis-type reversible enzyme:PBMX-R complex, before the formation of the covalent irreversible enzyme-PBMX-R adduct [36]. This indicates that the reaction obeyed pseudo-first-order saturation kinetics. The maximum inactivation rate (*k_3_*) and the dissociation constant (*K_D_*) were determined, and the results are listed in Table 1.

The selectivity of the interaction between hGSTA1-1 and PBMX-R was demonstrated by the competition observed between the enzyme’s inhibitor S-p-nitrobenzyl-glutathione (S-pNb-GSH) and the PBMX-R, as shown in Figure 3. In the presence of S-pNb-GSH (1 mM), the inactivation of the enzyme by PBMX-R is suppressed, suggesting a competition between the two ligands for binding at the same site on the enzyme. The inhibitor S-pNb-GSH is an S-substituted glutathione analog, in which the thiol hydrogen of glutathione was replaced by the 4-nitrobenzyl group. S-pNb-GSH binds to hGSTA1-1 and overlaps both the G- and H-site [2].

The stoichiometry of the hGSTA1-1-PBMX-R covalent complex was determined photometrically by measuring the amount of the dye conjugated to the enzyme. The results indicated that the inactivated enzyme contains 1.1 ± 0.1 mol of dye per mol of dimeric protein.

### 2.3. Kinetic Inhibition Analysis

**Figure 5:**
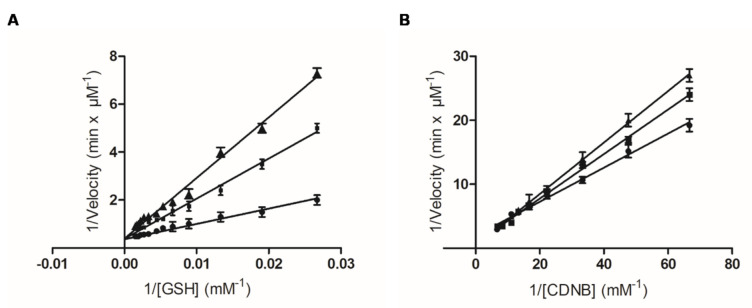
Kinetic inhibition analysis of the interaction of hGSTA1-1 with the hydrolysed PBMX-R (hPBMX-R). Lineweaver-Burk plots of the dependence of the catalytic reaction on substrate concentration in the presence or absence of different concentrations of hPBMX-R. (**A**) Inhibition of hGSTA1-1 by the hPBMX-R [(0 μM (●), 2 μM (■), 5 μM (▲)] at a constant CDNB concentration and variable GSH concentrations. (**B**) Inhibition of hGSTA1-1 by hPBMX-R [(0 μM (●), 2 μM (■), 5 μM (▲)] at a constant GSH concentration and variable CDNB concentrations. The data points represent mean value with error.

Kinetic inhibition analysis was carried out to investigate the type and the inhibition pattern of PBMX-R. Before the analysis, PBMX-R was converted to its monochlotriazine form (hPBMX-R). In hPBMX-R, one chlorine group of the triazine ring was substituted with a hydroxyl group (-OH). This substitution reduces the electrophilicity of the triazine ring [30], and as a consequence, hPBMX-R can function as a reversible inhibitor. We assume that that the small modification in the structure of PBMX-R does not significantly affect its binding mode with the enzyme. The Lineweaver–Burk plots of the dependence of the catalytic reaction on substrate concentration in the presence or absence of different concentrations of hPBMX-R are shown in Figure 5. The inhibition pattern obtained suggests that PBMX-R behaves as a linear competitive inhibitor towards GSH, with *K_i_* 2.02 ± 0.1 μΜ, and mixed-type inhibitor towards CDND, with *K_i_* 14.8 ± 1.1 μΜ.

### 2.4. The Identification of hGSTA1-1 Residue Was Modified by PBMX-R

**Figure 6:**
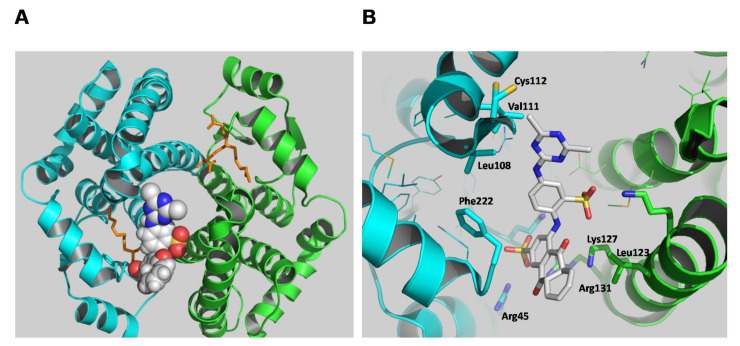
The predicted favorable binding mode of the PBMX-R with the hGSTA1-1 (PDB code 1k3l). (**A**) PBMX-R is shown as a space-filling model, and the inhibitor S-hexyl-glutathione is depicted in stick representation and colored dark yellow. (**B**) Important side chains that contribute to interaction are shown and labeled. Amino acid side chains and PBMX-R are depicted in stick representation and colored according to the atom type.

A molecular docking study was employed to predict the binding site of the PBMX-R. The most favorable mode of binding (FullFitness: −2090.22, deltaG: −9.97) is illustrated in Figure 6. The PBMX-R binds at a discrete site that is located at the entrance of the substrate-binding site and partially occupies the G-site. This mode of binding consists of the competitive type of inhibition towards GSH and mixed-type inhibition towards CDNB that was obtained by the kinetic inhibition analysis (Figure 5). The anthraquinone ring’s sulfonic group occupies the position of glycine carboxylate of GSH at the G-site and forms strong electrostatic interactions with Arg45 and Arg131. The side-chain of Lys127 forms an amino-aromatic interaction with the anthraquinone ring, and Leu123 provides a hydrophobic environment for the binding of anthraquinone. Phe222 seems to be able to form aromatic-aromatic interaction with the benzyl-sulfonate bridging ring of the PBMX-R. The triazine ring is oriented towards the Cys112, suggesting that the side-chain of Cys112 is the nucleophilic group that reacts with the electrophilic carbon on the triazine ring. Other residues, such as Leu108 and Val111, provide van der Waals contacts with the triazine group.

Cys112 is a solvent-exposed residue located at the loop (Figure 6) that links the H4 and H5 helices. It is involved in forming the large V-shaped cleft formed at the dimer interface, which previous studies showed that it contributes to the formation of L-site [8,36,37]. Previously published data have established that the side-chain of Cys112 is highly reactive and accessible for chemical modification [8,36,37]. For example, the reaction of the fluorescence ligand *N*-iodoacetyl-*N′*-(5-sulfo-1-naphthyl)ethylenediamine with hGSTA1-1 leads to modification of Cys112 [29,38]. The modification of Cys112 alters the affinity of the anionic dye 8-anilinonaphthalene 1-sulfonate (ANS) with the enzyme, suggesting that this residue participates in the formation of the ligandin binding site [38]. Therefore, it is conceivable to assume that the alkylation of Cys by a bulky compound (such as PBMX-R) blocks the cleft and impedes substrates from binding.

The modification of Cys112 appears to have a significant impact on the structure of the interface, the volume and the size of the solvent channel, the binding of GSH and substrate, and the product’s release. Kumari et al. 2016 have shown that the reaction hGSTA1-1 with phenyl isothiocyanate is achieved at Cys112. The modified enzyme shows more than two orders of magnitude inhibition in vitro [37].

To confirm whether a cysteine residue is involved in the chemical modification reaction, we determined the number of cysteine residues in the PBMX-R-modified and intact enzyme. Cys112 is the only cysteine residue in the sequence of hGSTA1-1 [31]. Total cysteine determination using the DTNB reagent showed that the hGSTA1-1-PBMX-R covalent adduct displayed a loss of 0.97 ± 0.21 mol of Cys/mol enzyme dimer, which is close to unity. The loss of cysteine after PBMX-R modification indicates that Cys112 is most probable the nucleophilic target for PBMX-R.

**Figure 7:**
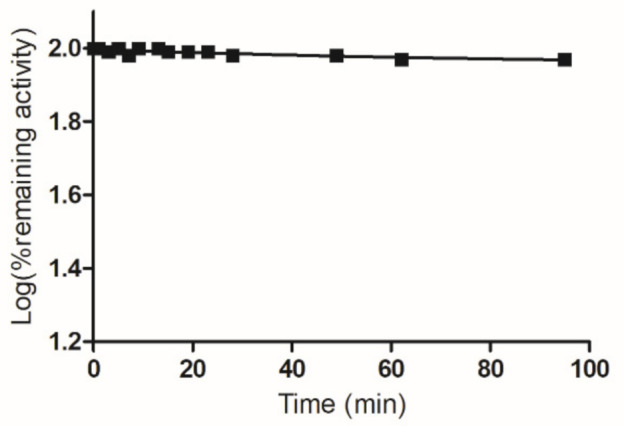
Time course of inactivation of maleimide-modified hGSTA1-1 by PBMX-R. The maleimide-modified hGSTA1-1 was incubated with 5.0 μM PBMX-R (■). At the times indicated, aliquots were withdrawn and assayed for enzymatic activity. The data points show one representative experiment.

To provide further experimental evidence and establish the involvement of Cys112 in the reaction with PBMX-R, chemical modification with maleimide was carried out. Lyon and Atkins have shown that Cys112 is modified by maleimide and the maleimide-modified enzyme retains most of its activity (92%) [31]. Incubation of the maleimide-modified enzyme with PBMX-R did not affect the remaining enzyme activity, indicating that the enzyme resistant to further modification by PBMX-R (Figure 7).

The biphasic kinetics observed (Figure 3) can be explained by assuming that the two subunits are not structurally equivalent. It is conceivable to assume that the reaction of PBMX-R with Cys112 in one subunit alters the susceptibility of Cys112, for chemical modification, in the other subunit, suggesting the existence of inter-subunit communication. This is supported by several pieces of evidence. For example, X-ray crystallography results have shown that the C-terminal region in the apo structure adopts different conformation in the two subunits [27]. Similar negative cooperativity occurs when hGSTA1-1 binds to the dinitrosyl–diglutathionyl iron complex [39].

**Figure 8:**
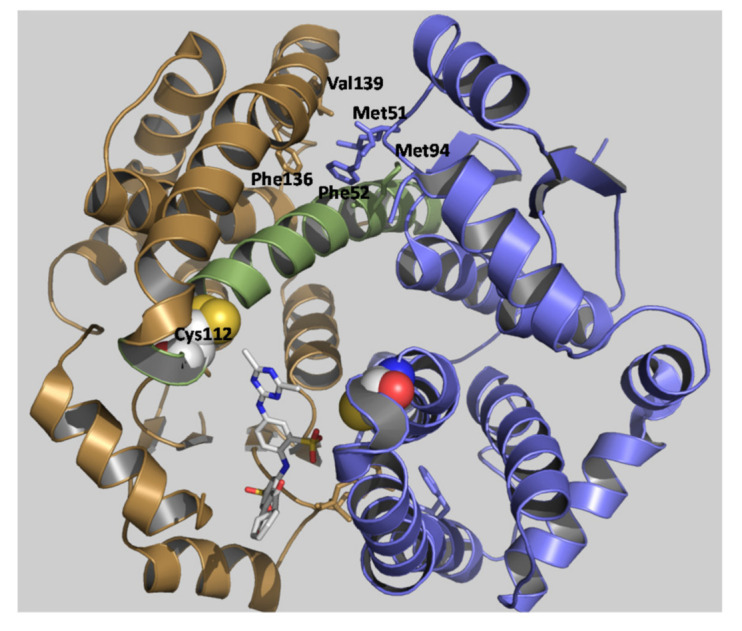
Predicted structural communication between hGSTA1-1 subunits (PDB code 1k3l). Amino acid residues that form the lock-and-key motif (Phe52, Met51, Phe136 and Val139) are shown in a stick representation and labeled. PBMX-R is shown in a stick representation and colored according to the atom type. α-Helix-4 is colored green. Cys112 is shown in sphere representation and colored according to the atom type.

The observed cooperativity is probable the consequence of the binding of the PBMX-R to the one subunit that alters the structure of the adjacent ligand-free subunit. Analysis of the subunit interface of hGSTA1-1 allows identifying the structural features that seem to contribute to the observed communication. Previous studies have established that the “lock-and-key” motif is a crucial structural element that significantly affects inter-subunit communication (Figure 8) [39,40]. This motif is formed by Phe52 and Met51 from one subunit that interacts with a hydrophobic patch on the other subunit, composed in part by Met94, Phe136, and Val139. A network of interactions allows a conformational change to be transmitted via the α-helix H4 to Cys112. As a result, this conformational change may restrict the accessibility of Cys112 at the second subunit for reaction with PBMX-R.

## 3. Materials and Methods

### 3.1. Materials

All reagents and chemicals were of analytical grade and obtained from Sigma-Aldrich Co (St. Louis, MO, USA).

### 3.2. Heterologous Expression and Purification of Recombinant Human Glutathione Transferase A1-1

The isoenzyme hGSTA1-1 was expressed in *E. coli* Bl21 (DE3) cells and purified by affinity chromatography on immobilized GSH as described previously [41]. Protein concentration was determined by the method of Bradford, using BSA as standard [42].

### 3.3. Assay of GST Activity and Inhibition Analysis by Triazine Dyes

Assay of GST activity was carried out using the CDNB/GSH system as described previously [41]. The inhibition of hGSTA1-1 by DCTDs was evaluated by measuring the ability of the compounds to bind and inhibit enzyme activity. The inhibition potency was measured with the CDNB/GSH assay system in the presence of 20 μΜ dye.

(1) $$\%I=100\frac{R_{o}-R_{i}}{R_{o}}$$

where R_o_ is the rate of absorbance increase for the uninhibited reaction and R_i_ is the rate of increase for the inhibited reaction. Both R_i_ and R_o_ correspond to the same substrate concentration.

Kinetic inhibition analysis was carried out as described previously [43]. Before kinetic analysis, the dichlorotriazine dye PBMX-R was converted to its monochlorotriazine form by hydrolysis [30,31]. The kinetic data were analyzed by nonlinear regression analysis using the computer program GraphPad Prism 5 software (San Diego, CA, USA).

### 3.4. Enzyme Inactivation Studies by PBMX-R

Inactivation of hGSTA1-1 was performed in 1 mL of incubation mixture containing: potassium phosphate buffer pH 7 (20 mM), PBMX-R, (0–10.0 μM); enzyme, (0.1 units). The inactivation rate was followed by periodically removing samples (5–20 μL) for assay of enzymatic activity. The chemical reaction between an enzyme and an irreversible inhibitor, such as PBMX-R, can be described as follows:

(2) $$E+\mathrm{PBMX}-R\;\overset{K_{D}}{⇄}\;E:\mathrm{PBMX}-R\;\overset{k_{3}}{\to }\;E-\mathrm{PBMX}-R+L$$

where E and PBMX-R are the free enzyme and the irreversible inhibitor, respectively, E:PBMX-R is the intermediate, Michaelis-type enzyme:inhibitor reversible complex, E-PBMX-R is the covalently modified enzyme by the inhibitor, *K_D_* is the dissociation constant of the complex E:PBMX-R, *k*_3_ is the maximum rate of inactivation and L is the reaction’s leaving group. By assuming Briggs and Haldane conditions and excess of inhibitor over enzyme concentration ([I] >> [E]), the kinetic equation describing the interaction is given by the following formula [30,31,44,45]:

(3) $$k_{obs}=\frac{k_{3}[I]}{K_{D}+[I]}$$

where *k_obs_* is the observed rate of inactivation of the enzyme by the non-reversible inhibitor, and [I] is the concentration of the non-reversible inhibitor. The observed rate of enzyme inactivation (*k_obs_*) for each concentration of PBMX-R was determined from the curves of the log(% remaining enzymatic activity) versus time t (min) at 25 °C. Rate constants for the reaction exhibiting biphasic kinetics were calculated as described previously [8,31,44,45,46] using the computer program GraphPad Prism 5 software (San Diego, CA, USA).

Inactivation studies of hGSTA1-1 by PBMX-R in the presence of S-pNb-GSH were performed in 1 mL of incubation mixture containing: potassium phosphate buffer pH 7 (20 μmol); PBMX-R, (5 μM); S-pNb-GSH (1 mM); enzyme (0.1 units).

The stoichiometry of PBMX-R/hGSTA1-1 conjugate was measured photometrically at 620 nm as described earlier [30].

### 3.5. Modification of hGSTA1-1 with DTNB and N-ethylmaleimide

Determination of enzyme cysteine-SH groups with 5,5-dithio-bis-(2-nitrobenzoic acid) was achieved using Ellman’s method [47] as described by Karpusas et al., 2013 [8]. Modification of hGSTA1-1 by N-ethylmaleimide (1 mM) was carried out according to Lyon and Atkins (2002) [36].

### 3.6. The Interaction of PBMX-R with hGSTA1-1 by Molecular Docking

The putative binding sites of PBMX-R were identified by molecular-docking using the SwissDock program and EADock DSS software [48,49]. The crystal structure of hGSTA1-1 with PDB code 1k3l (1.5 Å resolution) was used for docking [50]. The docking was performed with no region of interest defined (blind docking). CHARMM energies were measured, and binding modes are evaluated using FACTS and clustered [51]. For inspection of models and crystal structures, the program PyMOL (http://www.pymol.org/ (accessed on 17 April 2021)) was used.

## 4. Conclusions

The present study showed that the dichlorotriazine dye PBMX-R is an irreversible alkylating inhibitor for hGSTA1-1. Considering that hGSTA1-1 contributes significantly to developing multidrug resistance to many anticancer drugs, the results suggest that PBMX-R is a useful probe suitable for assessing by kinetic means the drugability of the enzyme in future drug-design efforts. The outcome of the study can facilitate a more rational development of effective GST-targeted cancer chemosensitizers.

## Figures and Tables

**Table 1:** Kinetic parameters of the fast and slow phase of hGSTA1-1 inactivation by PBMX-R.

Parameter	Fast	Slow
*K_D_*	5.2 ± 0.5	3.4 ± 0.2
*k*_3_	1.3 ± 0.1	0.06 ± 0.003

## Data Availability

Data is contained within the article.

## Abbreviations

CB3GA	Cibacron blue 3GA
CDNB	1-chloro-2,4-dinitrobenzene
DCTD	dichlorotriazine dye
GSH	glutathione
GST	glutathione transferase
G-site	glutathione-binding site
hGSTA1-1	human glutathione transferase A1-1
hPBMX-R	hydrolyzed PBMX-R
H-ste	hydrophobic binding site for electrophilic substrates
PBMX-R	Procion blue MX-R
L-site	ligandin-binding site; (L-site)
S-pNb-GSH	S-p-nitrobenzyl-glutathione
